# CD274/PD-L1 copy number gained malignant peripheral nerve sheath tumor: A case report and literature review

**DOI:** 10.1097/MD.0000000000041165

**Published:** 2025-01-03

**Authors:** Kiyong Na, Hong Jun Kim

**Affiliations:** a Department of Pathology, College of Medicine, Kyung Hee University Hospital, Seoul, Korea; b Department of Medical Oncology, College of Medicine, Kyung Hee University Hospital, Seoul, Korea.

**Keywords:** case report, CD274/PD-L1, genomic profile, immune checkpoint inhibitor, malignant peripheral nerve sheath tumor, next-generation sequencing

## Abstract

**Rationale:**

Malignant peripheral nerve sheath tumors (MPNSTs) are aggressive soft tissue sarcomas with a poor prognosis, particularly in metastatic cases. Traditional treatments have shown limited effectiveness, highlighting the need for innovative therapeutic approaches. This case report aims to emphasize the critical role of genomic profiling in identifying therapeutic targets, particularly immune checkpoint inhibitors, to improve treatment strategies for MPNST.

**Patient concerns:**

An 82-year-old male presented with a long-standing history of MPNST, multiple recurrences, and a recent rapid enlargement of a mass in the right axillary region. The patient also reported a 10% weight loss over the last 6 months.

**Diagnoses:**

Comprehensive genomic profiling of the tumor revealed significant alterations, including CD274/PD-L1 amplification, CDKN2A loss, and TP53 mutation. These genetic findings were aligned with previous cases that responded favorably to immune checkpoint inhibitors.

**Interventions:**

Despite the potential for targeted immunotherapy, the patient’s economic constraints prevented the initiation of immune checkpoint inhibitor therapy. The patient underwent multiple surgical interventions, including an above-elbow amputation.

**Outcomes:**

The patient experienced severe wound bleeding and a significant decline in general condition, requiring intensive care unit support. Given the poor prognosis and high surgical risks, the patient’s caregivers opted for hospice care.

**Lessons:**

Genomic profiling identifies genetic alterations that could guide immune checkpoint inhibitor therapy, offering the promise of personalized treatment for MPNST patients. By highlighting the potential of genomic profiling, this case demonstrates the importance of integrating personalized immunotherapy into future treatment paradigms for MPNST.

## 1. Introduction

Malignant peripheral nerve sheath tumors (MPNSTs), a rare subset of aggressive soft tissue sarcomas (STS), present a significant clinical challenge. These tumors arise from the sheaths of peripheral nerves and are associated with a grave prognosis. Metastatic cases are particularly dire, with a 3-year survival rate of only 14%.^[1]^ Patients with neurofibromatosis type 1 (NF1) who transition from benign neurofibromas to malignant MPNSTs typically have a worse prognosis than those with sporadic tumors.^[2]^ This underscores the urgent need for improved diagnostic methods and innovative therapeutic strategies to enhance survival rates.

The standard treatment for MPNST primarily involves surgical resection with adjuvant radiation, targeting localized high-grade cases.^[3]^ Despite these efforts, many patients still suffer from local recurrences or distant metastases.^[4]^ While complete surgical excision with clear margins provides the best chance for a cure, particularly in tumors >5 cm, adjuvant treatments such as radiotherapy and cytotoxic chemotherapy have limited success in prolonging survival.^[3–5]^ In cases of metastatic disease, treatment options are largely palliative, emphasizing the critical need for more effective therapeutic approaches.^[5]^

Molecular profiling has revealed the complex genetic landscape of MPNST, identifying frequent mutations and dysregulations in signaling pathways.^[6]^ Despite these insights, targeted therapies have generally not met expectations in terms of clinical effectiveness.^[7]^ The low expression of PD-L1 in MPNST,^[8]^ contrasted with a relatively high presence of cytotoxic T cells, suggests a potential for immune checkpoint inhibition.^[9]^ This emerging area, highlighted by promising results in other malignancies, indicates that immunotherapy could represent a new therapeutic avenue for MPNST, even without established treatment protocols that include these agents.

To date, 4 case reports have documented MPNSTs responding favorably to immune checkpoint inhibitors.^[10–13]^ We report the case of an 82-year-old Asian male with metastatic MPNST, characterized by an increase in the number of CD274/PD-L1 copies.

## 2. Case description

An 82-year-old male with a significant medical history, including chronic kidney disease, hypertension, atrial fibrillation, chronic obstructive pulmonary disease, and a previous cerebrovascular accident, presented with a rapidly enlarging mass in the right axillary region, noticed just a few weeks prior. He also reported a 10% weight loss over the last 6 months. The patient had been battling undifferentiated pleomorphic sarcoma of the right forearm, first diagnosed 6 years ago. Over the years, he underwent multiple surgical interventions, including 7 wide excisions to manage recurrent disease. Four months ago, he underwent an above-elbow amputation of the right arm due to uncontrollable bleeding. The initial forearm tumor was characterized as an expansile mass with a well-demarcated border, rated as French Federation of Cancer Centers Sarcoma Group grade 3/3 for differentiation, with moderate tumor necrosis (1/2), and a high mitotic count (3/3). The pathological TNM staging was classified as pT3Nx according to the American Joint Committee on Cancer 8th edition.

**Figure 1.**
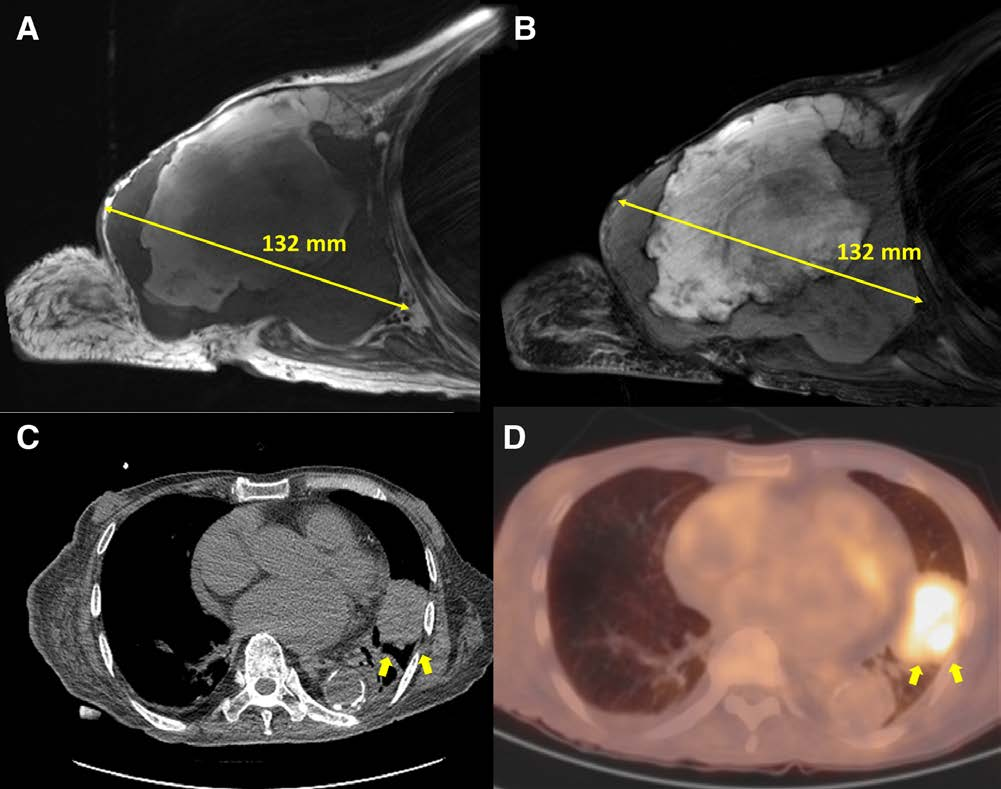
Primary tumor and metastatic lung mass. (A) T1-weighted magnetic resonance imaging (MRI) showing a well-defined soft tissue mass located between the amputated proximal humerus and the right hemithorax, measuring 13.2 × 12.5 cm. (B) T2-weighted MRI depicting a soft tissue mass with heterogeneous enhancement. (C) Computed tomography (CT) imaging illustrating a large lobulated mass in the left upper lobe lingular segment. (D) Positron emission tomography-computed tomography (PET-CT) scans revealing a mass in the lingular segment of the left lung.

Upon physical examination, a fixed, non-tender mass was palpable in the right axillary area. Laboratory findings revealed a hemoglobin level of 8.9 g/dL, a white blood cell (WBC) count of 11,670/µL, platelets at 135,000/µL, AST of 21 U/L, ALT of 9 U/L, total bilirubin of 0.58 mg/dL, BUN of 21 mg/dL, creatinine of 1.33 mg/dL, sodium at 138 mmol/L, potassium at 3.8 mmol/L, and chloride at 106 mmol/L. Subsequent magnetic resonance imaging revealed a large, well-defined soft tissue mass in the right axilla, measuring 13.2 × 12.5 × 16.7 cm. The mass showed heterogeneous high signal intensity on T1 and T2-weighted images and displayed mild post-contrast enhancement, with no evidence of fat within the lesion. Positioned between the amputated proximal humerus and the right hemithorax, the mass extended inferiorly to the skin, suggesting recurrent sarcoma (Fig. 1). Further imaging with chest computed tomography and positron emission tomography-computed tomography scans identified additional potential metastatic sites, including subpleural and lingular lesions in the left lung, as well as lesions in the sternum and T11 vertebra (Fig. 1).

Given the diagnosis of stage IV sarcoma and the associated risks of significant bleeding, a palliative-wide excision of the axillary mass was performed to manage symptoms. The surgery successfully removed the mass, significantly alleviating compression symptoms. Postoperatively, the patient’s hemoglobin stabilized, and wound healing progressed without immediate complications. Histopathological examination confirmed the recurrence of an undifferentiated pleomorphic sarcoma, reclassified as a high-grade MPNST according to the 5th edition of the World Health Organization classification. This reclassification was supported by SOX-10 and H3K27me3 immunohistochemistry findings. The tumor, measuring 22 × 17 × 9 cm, was characterized as an expansile mass with an infiltrative border, a French Federation of Cancer Centers Sarcoma Group grade of 3/3, extensive necrosis (2/2), and a high mitotic count (3/3). SOX-10 showed patchy positivity, and H3K27me3 staining was <5%. Molecular profiling was conducted using targeted next-generation sequencing (NGS) (Oncomine Comprehensive Assay v3, Thermofisher Scientific, Waltham, MA). This analysis revealed significant genetic alterations: a TP53 mutation characterized by a missense change (p.R273H, c.818G>A) with a 53% allele frequency, a TET2 mutation presenting as a nonsense mutation (p.R544*, c.1630C>T) with a 6% allele frequency (Table 1), major copy number variations including CD274 amplification at an 11.5 copy number, and a significant deletion in CDKN2A with a copy number of 0.18 (Table 2). Additionally, the analysis indicated a loss of heterozygosity at 33.3%, microsatellite stable status with a microsatellite instability score of 0.82, a tumor mutation burden of 1.9 mutations/Mb, and no detected fusion or splice variants. The final diagnosis of the presented case is CD274/PDD-L1 copy number gained MPNST.

**Table 1:** Molecular alterations confirmed by next generation sequencing in malignant peripheral nerve sheath tumor containing about 50% tumor cells.

Gene	Mutation type	Amino acid change	Sequence change	Allele frequency	Pathogenicity
TP53	Missense	p.R273H	c.818G>A	53%	Pathogenic
TET2	Nonsense	p.R544*	c.1630C>T	6%	Pathogenic

**Table 2:** Major copy number variations confirmed by next generation sequencing in malignant peripheral nerve sheath tumor.

Region	Gene	Type of variation	Copy number
9p24.1	PDCD1LG2	Amplification	10.7
9p24.1	CD274	Amplification	11.5
9p21.3	CDKN2A	Deletion	0.18
9p21.3	CDKN2B	Deletion	0
6p22.1	HLA-A	Deletion	0.35
17q12	RAD51D	Deletion	0
9p21.3	MTAP	Deletion	0.07

Initially deemed suitable for anthracycline-based chemotherapy upon his diagnosis of stage IV MPNST, the patient’s rapidly deteriorating health soon made cytotoxic chemotherapy infeasible. The tumor board recommended immune checkpoint inhibitors based on a favorable response to anti-PD1 treatment observed in another case with a similar genomic profile.^[13]^ However, due to financial constraints, the patient declined further interventions, including systemic treatment.

Three months later, he was readmitted for surgical management of severe wound bleeding but quickly experienced a significant deterioration in his general condition and desaturation, necessitating intensive care unit support. Despite aggressive supportive measures, the patient’s condition did not improve, and hospice care was initiated. Hospice interventions focused on pain control and maintaining the patient’s comfort. He was subsequently discharged with a comprehensive palliative care plan.

## 3. Discussion

This case, identified through NGS, displays a distinct MPNST subtype characterized by CD274/PD-L1 amplification, CDKN2A loss, TP53 mutation, and TET2 mutation. Although no treatment was administered, these findings are particularly noteworthy as they align with a previous report,^[13]^ in which a similar molecular subtype showed a remarkable response to immune checkpoint inhibitor therapy. This correlation highlights the potential for targeted immunotherapeutic responsiveness in MPNST subsets with specific genetic profiles. Previously documented cases,^[10–13]^ totaling 4, have demonstrated varying degrees of therapeutic success using such inhibitors (Table 3). These instances include a patient with NF-1 mutated MPNST achieving complete remission with pembrolizumab,^[12]^ another showing significant improvement post-radiotherapy with the same agent,^[10]^ a third case where pembrolizumab combined with procarbazine provided therapeutic benefits,^[11]^ and a fourth illustrating a sustained response with nivolumab alongside radiotherapy.^[13]^ These examples underscore the evolving understanding and potential therapeutic implications of immunotherapy in the management of MPNSTs despite the current absence of FDA approval for this specific indication.

**Table 3:** Four cases of malignant peripheral nerve sheath tumors with favorable responses to immune checkpoint inhibition.

Ref	Sex (age)	Treatment	Result	Biomarker
^[10]^	M(22)	Radiotherapy 54Gy Pembrolizumab 4C	CR	PD-L1 5% (IHC)
^[11]^	M(48)	Doxorubicin plus Ifosfamide Imatinib Eribulin Pembrolizumab 6C plus procarbazine	CR	PD-L1 90% (TPS)
^[13]^	M(45)	Surgical resection Doxorubicin 2C Ifosfamide 2C Nivolumab 12C plus radiotherapy	Deep and prolonged response	CD274/PD-L1 amplification
^[12]^	M(60)	Debulking surgery Epirubicin plus ifosfamide 3C Pembrolizumab 5C	CR	PD-L1 70% (IHC)

C = cycle; CR = complete remission; IHC = immunohistochemistry; TPS = tumor proportion score.

MPNSTs are aggressive, poorly differentiated neoplasms originating from the Schwann cell lineage, accounting for approximately 5% to 10% of all STSs.^[3]^ Around 40% to 50% of MPNSTs develop in patients with NF1, while sporadic cases constitute another 40% to 47%. The remainder often arise at sites of previous radiotherapy.^[14]^ NF1 is characterized by the growth of both benign and malignant peripheral nerve sheath tumors, which can lead to significant morbidity by compressing vital structures and potentially transforming into MPNSTs.^[15]^ The only curative treatment for MPNSTs is complete surgical resection with wide negative margins, although this is often complicated by factors such as the tumor’s size, location, or the presence of metastases.^[7]^ Radiotherapy is typically employed to enhance local control in patients harboring large high-grade tumors or positive resection margins but has shown no impact on overall survival.^[16]^ Evidence supports that neoadjuvant chemotherapy, particularly with agents like doxorubicin and ifosfamide, might render some locally advanced primary MPNSTs operable, thereby improving disease-free survival.^[17]^ Despite these interventions, the objective response rates to chemotherapy in advanced MPNST remain dismally low, between 20% and 30%.^[18]^ Consequently, there are no definitive effective treatment options for patients with recurrent, unresectable, or metastatic disease, underscoring the urgent need for these patients to participate in clinical trials exploring new targeted therapies. Unfortunately, the efficacy of targeted, noncytotoxic treatments in MPNSTs has been limited, typically achieving stable disease in fewer than 25% of patients, and with a median progression-free survival of <2 months.^[19]^ These challenges underscore the need to expand our exploratory scope to include various therapeutic strategies, notably immunotherapy, in managing MPNSTs. This approach is vital for developing more effective therapeutic strategies, which could substantially alter the clinical outcomes for patients afflicted with this aggressive form of cancer.

In the landscape of STS, the efficacy of immune checkpoint therapy varies widely, with PD-L1 expression often guiding the likelihood of response.^[20]^ Unlike other STS subtypes where traditional treatments like doxorubicin remain standard, studies have shown that immune checkpoint inhibitors can have markedly different outcomes.^[20]^ For instance, a phase II trial demonstrated an objective response rate of 18% in STS to pembrolizumab, highlighting varied responses across subtypes such as undifferentiated pleomorphic sarcoma, liposarcoma, synovial sarcoma, and leiomyosarcoma, with notably low responsiveness in bone sarcomas like osteosarcoma and chondrosarcoma.^[21]^ Similarly, clinical trials evaluating nivolumab^[22]^ and ipilimumab^[23]^ for STS have demonstrated limited efficacy. MPNST, deriving from neural crest cells, exhibits a unique response pattern to immune checkpoint inhibitors, distinct from other STS subtypes. This differentiation potentially stems from their neural crest origins, paralleling the success seen in melanoma, another neural crest cell-derived malignancy, where PD-1 blockade has significantly improved survival outcomes in numerous clinical trials.^[24]^ MPNST cases reviewed in our study show a remarkable response rate under immune checkpoint inhibitors. The current understanding of immune checkpoint inhibitors in MPNST versus other STS highlights the need for further research, particularly to elucidate the mechanisms behind the varying efficacy of immunotherapy across different sarcoma subtypes. Upcoming results from ongoing randomized controlled trials such as NCT02834013 and NCT02721732, which include rare tumors like malignant adrenal pheochromocytomas and paragangliomas derived from neural crest cells, are anticipated to provide deeper insights. These studies aim to further our understanding of the predictors of ICT sensitivity, including PD-L1 expression and specific genetic markers, potentially leading to more tailored and effective treatment strategies for patients with these challenging malignancies.

The outcomes of PD-1/PD-L1 inhibitors in treating various STS have been variably effective yet promising.^[21,25,26]^ The exploration of immune checkpoint inhibitors in MPNST is further illustrated by 3 phase II trials. The first study (NCT02691026) evaluates the effect of pembrolizumab on unresectable or metastatic MPNST, aiming to assess clinical responses over ten treatment cycles. The second trial (NCT02834013) investigates the combination of nivolumab and ipilimumab in rare tumors, including MPNST, to determine overall response rates and explore the potential of these therapies in PD-L1 amplified cancers. Lastly, NCT03611868 examines the efficacy of APG-115 combined with pembrolizumab, focusing on dose escalation and response in MPNST and melanoma patients. These trials highlight the growing interest in the role of immunotherapy against MPNST and promise new insights into effective treatment strategies.

Emerging in vitro studies illuminate the immune landscape of MPNST, offering nuanced insights into PD-L1 expression and immune cell infiltration.^[9,27]^ Although direct evidence from clinical trials is still forthcoming, these studies suggest a potential avenue for ICI therapy in MPNST, particularly given the observed variability in PD-L1 expression and the presence of CD8+ TILs.^[9]^ Elevated PD-L1 levels in MPNST patients, as demonstrated in some analyses, along with the identification of immune modulatory factors, underscore a potential predictive role for these markers in ICI responsiveness.^[28]^ This foundational knowledge supports a deeper investigation into the immunobiology of MPNST, encouraging future studies to explore the therapeutic potential of ICIs in this challenging malignancy.

In immuno-oncology, identifying effective predictive biomarkers is crucial yet challenging. While PD-L1 overexpression is widely used, its limitations are apparent. In contrast, CD274/PD-L1 gene amplification emerges as a promising marker, notably for its prevalence and the associated high response rates to anti-PD-1 therapy in classic Hodgkin lymphoma (97%).^[29,30]^ Interestingly, approximately 30% of cases show no correlation between PD-L1 expression and gene amplification.^[31]^ CD274/PD-L1 amplification, variably observed across many solid tumor types, was identified in 11% of MPNST cases within The Cancer Genome Atlas, highlighting its potential to refine treatment strategies for these complex cancers.^[32,33]^ Additionally, the 9p24 locus, which encompasses both CD274/PD-L1 and JAK2, is crucial as JAK2 activation enhances PD-L1 transcription via increased STAT protein secretion, influencing responses to anti-PD-1 therapies.^[34]^ Preclinical studies also suggest that mutations in the JAK/STAT pathway may inhibit response to interferon γ receptor signaling, potentially contributing to resistance against anti-PD-1 treatments.^[35–37]^

Limitations of this study include its focus on a single case, which may not fully represent the variability seen in MPNSTs. Additionally, the absence of experimental validation for identified genomic alterations limits our ability to confirm their functional roles. The unavailability of direct treatment for this patient further restricts the conclusions that can be drawn about the clinical utility of immune checkpoint inhibitors for this genetic profile.

## 4. Conclusion

This case intriguingly shares a specific genomic profile (CD274/PD-L1 amplification, CDKN2A loss, and TP53 mutation) with a previous report where a patient demonstrated a significant response to immune checkpoint inhibitors.^[13]^ Although treatment was not pursued due to economic constraints, as NGS becomes increasingly accessible, there is significant potential to identify patient groups likely to benefit based on specific genomic characteristics. Given the limitations of PD-L1 as a predictive biomarker, it is imperative to further investigate the role of CD274/PD-L1 amplification in MPNST as a potentially significant predictive biomarker in immuno-oncology.

## Author contributions

**Conceptualization:** Hong Jun Kim.

**Writing – original draft:** Kiyong Na.
